# Is There an Interspecific Diversity of the Thymic Microenvironment?

**DOI:** 10.1155/1993/48056

**Published:** 1993

**Authors:** Lucia Renata Meireles de Souza, Valeria Trajano, Wilson Savino

**Affiliations:** Department of Immunology Institute Oswaldo Cruz-FIOCRUZ Rio de Janeiro Brazil

**Keywords:** Mammalian thymuses, thymic microenvironment heterogeneity, cytokeratins, thymic epithelium, extracellular matrix

## Abstract

Thymic epithelial cells (TEC) heterogeneity suggests the existence of functional subsets. Anti-cytokeratin (Anti-CK) monoclonal antibodies (MAb), markers of epithelial differentiation, have been used to detect TEC subsets in rodents and humans. These MAb revealed a different topography of CK-defined TEC subsets in mice and humans, leading us to carry out a comparative study of mammalian thymuses. Our study showed that the distribution pattern of cytokeratins in the thymic epithelium is complex and unique, with coexpression of CK typical of simple and stratified epithelia. Moreover, we demonstrated an interspecific diversity of CK expression within the thymic lobules. Interestingly, such diversity was not a general phenomenon for the expression of any thymic microenvironmental proteins, because the location of extracellular matrix components was essentially similar in the mammalian species studied.


*Developmental Immunology*, 1993, Vol. 3, pp. 123–135
Reprints available directly from the publisher
Photocopying permitted by license only

© 1993 Harwood Academic Publishers GmbH
Printed in the United States of America

### Is There an Interspecific Diversity of the Thymic Microenvironment?

LUCIA RENATA MEIRELES DE SOUZA, VALERIA TRAJANO, and WILSON SAVINO*

*Department of Immunology, Institute Oswaldo Cruz–FIOCRUZ, Rio de Janeiro, Brazil*

Thymic epithelial cells (TEC) heterogeneity suggests the existence of functional subsets. Anti-cytokeratin (Anti-CK) monoclonal antibodies (MAb), markers of epithelial differentiation, have been used to detect TEC subsets in rodents and humans. These MAb revealed a different topography of CK-defined TEC subsets in mice and humans, leading us to carry out a comparative study of mammalian thymuses. Our study showed that the distribution pattern of cytokeratins in the thymic epithelium is complex and unique, with coexpression of CK typical of simple and stratified epithelia. Moreover, we demonstrated an interspecific diversity of CK expression within the thymic lobules. Interestingly, such diversity was not a general phenomenon for the expression of any thymic microenvironmental proteins, because the location of extracellular matrix components was essentially similar in the mammalian species studied.

KEYWORDS: Mammalian thymuses, thymic microenvironment heterogeneity, cytokeratins, thymic epithelium, extracellular matrix.

### INTRODUCTION

Intrathymic lymphocyte differentiation events, including the selection of the T-cell repertoire, essentially occurs in the context of the so-called thymic microenvironment (Sprent et al., 1988; von Boehmer et al., 1989; Fowlkes and Pardoll, 1989; Blackman et al., 1990; Boyd and Hugo, 1991). As such microenvironment is rather complex—being composed mostly by the thymic epithelial-cell network together with other stromal cells and extracellular matrix—the precise role of each nonlymphoid component on the general process of thymocyte maturation remains to be determined. Thus, the characterization of these components would be helpful to further study the interactions between these thymic compartments, including using *in vitro* systems. Considering that thymic epithelial cells (TEC) pleiotropically influence steps of T-cell maturation (Kyewski, 1986; Schuurman, 1988; Haynes, 1990; van Ewijk, 1991), and that these cells are morphologically and antigenically heterogenous (Haynes, 1984; von Gaudecker, 1986; Kendall, 1988), one can imagine that TEC subsets might be involved in specific events of intrathymic T-cell differentiation. Yet, functionally defined TEC subsets were not isolated so far, despite the several TEC lines (Itoh, 1979; Nieburgs et al., 1985; Potworowski et al., 1986; Mizutani et al., 1987; Naquet et al., 1989) and the variety of available monoclonal antibodies (MAb) that are phenotypic markers of so-called TEC subsets (Haynes, 1984; van Vliet et al., 1984; de Maagd et al., 1985; Kaneshima et al., 1987; Takacs et al., 1987; Izon and Boyd, 1990). In this respect, a nomenclature for these anti-TEC MAb, subdividing them on the basis of their labeling patterns in 5 main groups called CTES—Clusters of Thymic Epithelial Staining—was proposed (Kampinga et al., 1989). Interestingly, these markers revealed a close antigenic similarity between the thymic epithelium and epidermis, with Hassall's corpuscles corresponding to a final degree of TEC differentiation, as the *stratum corneum* keratinocytes (Haynes, 1984; Schmitt et al., 1987). In this context, anticytokeratin (anti-CK) MAb, epithelial differentiation markers, might be regarded as useful tools in the study of TEC subsets, as actu-

*Corresponding author.

ally evidenced in some rodents (Nicolas et al., 1985, 1986; Colic et al., 1988a, 1988b; Savino and Dardenne, 1988a; Farr and Brady, 1989) and humans Savino and Dardenne, 1988b). Nonetheless, the location of a given CK-defined TEC subset within the thymic lobules was different in mouse as compared to man (Savino and Dardenne, 1988a, 1988b), suggesting a species-specific diversity of CK expression in mammalian thymuses. These data prompted us to carry out a comparative study of CK-defined TEC subsets in mammals. This analysis actually showed an interspecies heterogeneity in the intrathymic location of CKs, including in early stages of thymus ontogeny (Meireles de Souza and Savino, 1993). Differently, such diversity was not observed regarding the expression of extracellular matrix proteins, herein applied as representatives of another thymic microenvironmental component. Additionally, the present work demonstrated that the pattern of CK expression by the thymic epithelium is complex and rather unique, with cytokeratins typically found in simple or stratified epithelia being coexpressed.

### RESULTS

### Detection of the Thymic Epithelial Cell network by Pan-Specific Anti-CK Antibodies

We analyzed the thymuses of mammals representatives of different phylogenetic branches, as seen in Fig. 1. In all species studied, both polyclonal and monoclonal antibodies pan-specific

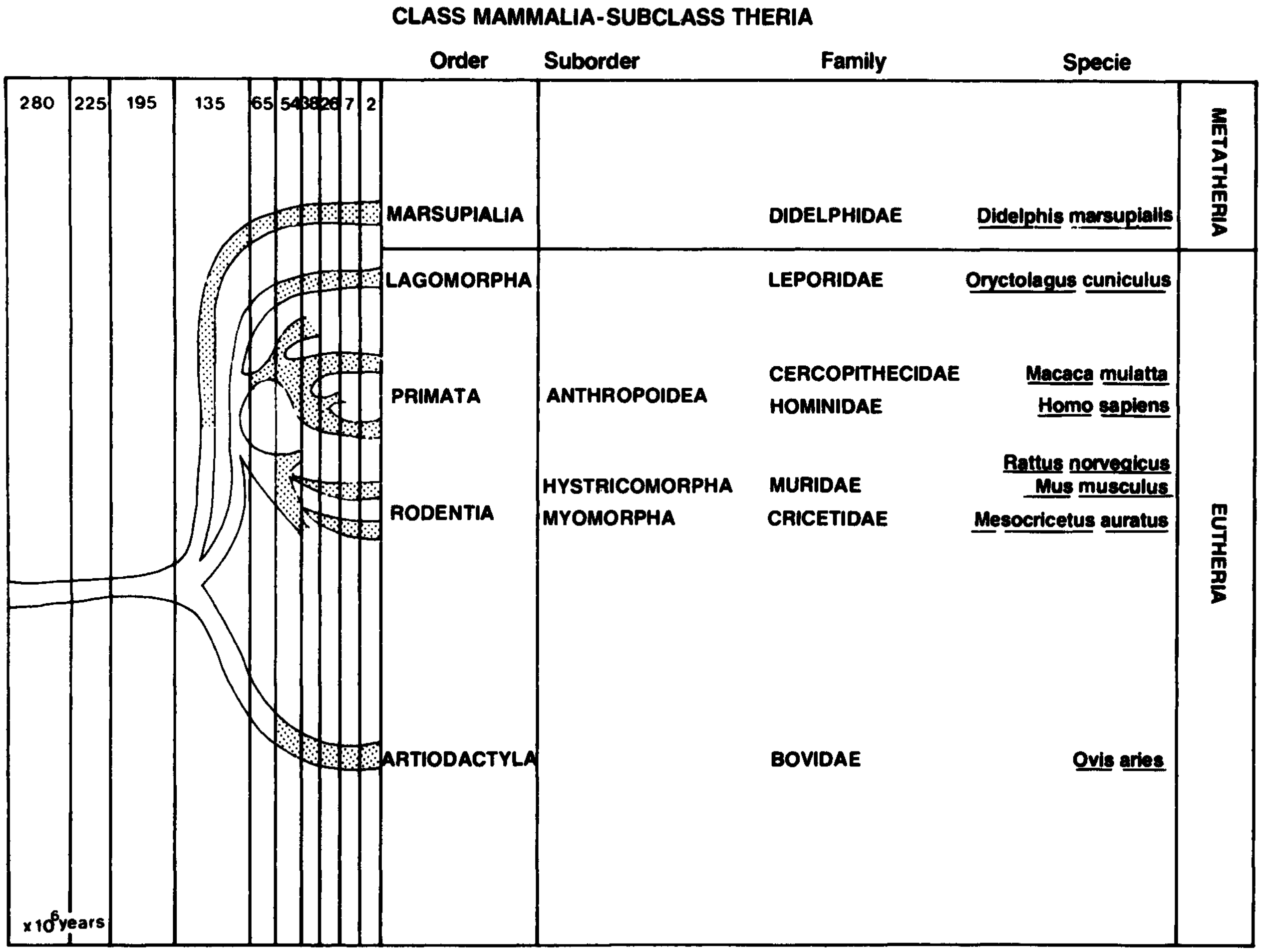

FIGURE 1. Classification of mammalian species studied. Open area: fossil data; shaded area: possible lines of descendence. Based on MacDonald (1984).

for cytokeratins (HTK and KL4, respectively) stained the whole network of epithelial cells, distinguishing the cortical and medullary TEC, these latter being more voluminous. Moreover, subcapsulary–subseptal TEC were evidenced (data not shown).

### Topography of TEC Expressing Cytokeratins Typical of Simple Lining Epithelia

In a first set of experiments, we studied the expression of CK8 and CK18 (strictly found in simple epithelia) and CK19, mainly detected in this type of epithelial tissues (Quinlan et al., 1985; Sun et al., 1985). In all cases, an interspecific diversity in CK distribution was seen. In primate thymuses, $CK8^+$ TEC were only observed in the medulla, a pattern exclusively found in this mammalian order. In contrast, the cortical region of rodent thymuses was consistently labeled by the anti-CK8 MAb, besides the medullary $CK8^+$ TEC in rat. Similarly, the rabbit thymic epithelium was also stained by the anti-CK8 MAb in both thymic regions. Differently, $CK8^+$ TEC were restricted to the cortico-medullary junction in the sheep thymus, and could not be detected in opossum thymus sections (Fig. 2).

Although CK8 and CK18 are a naturally occurring pair, their colocalization was solely integral in the primate and rodent orders. The major disparity of CK8 and CK18 immunodetection was found in the opossum thymus, because it was $CK8^-$, being entirely labeled by the anti-CK18 MAb.

Regarding the intrathymic distribution of CK19, we also evidenced an interspecific diversity in all orders studied. Rabbit and opossum TEC networks were virtually CK19 negatives, whereas the thymic epithelium of sheep, hamster, and human were completely stained. In the other mammalian species tested, $CK19^+$ TEC were mostly medullary (Fig. 3).

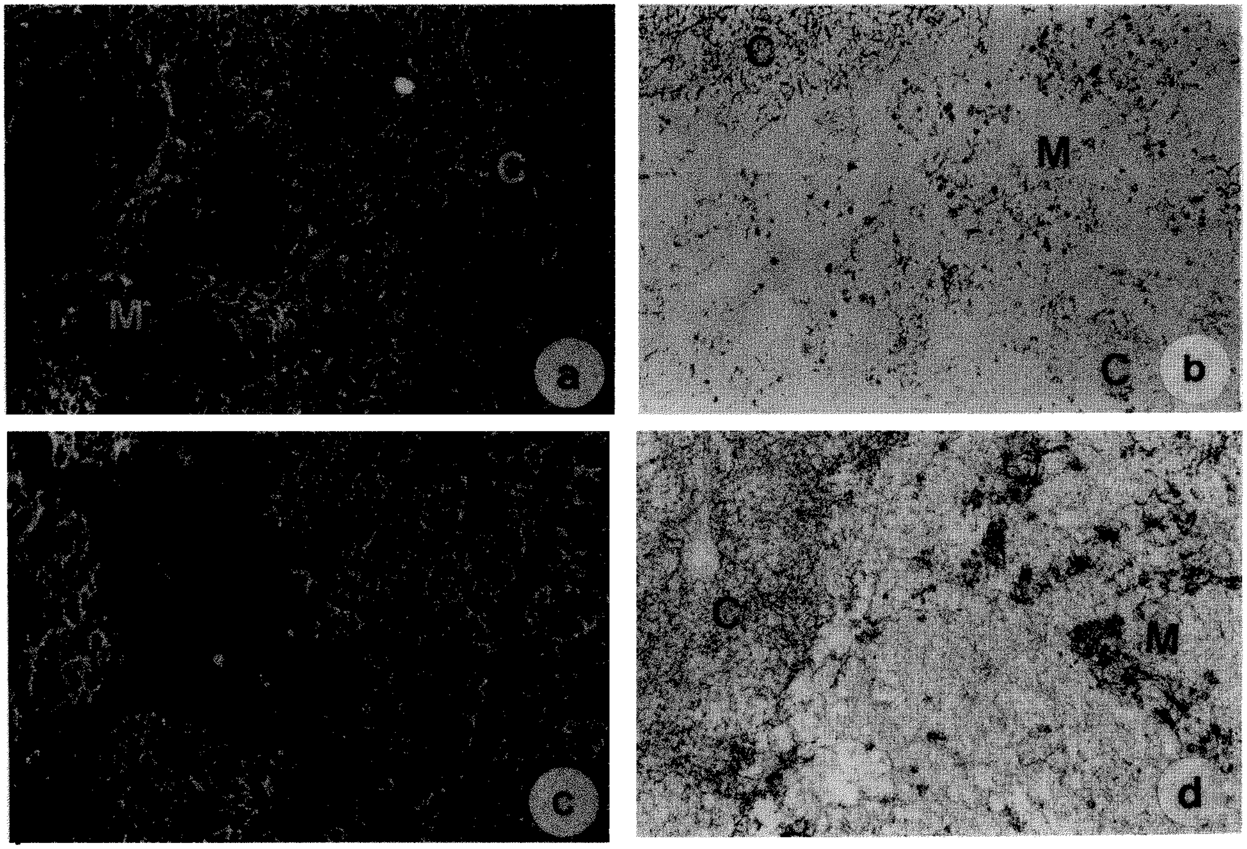

FIGURE 2. CK8/18-expression pattern in mammalian thymuses: (a) rat thymus section, showing the whole TEC network stained by the anti-CK8 MAb (× 200); (b) $CK8^+$ cortical zone of rabbit thymus (× 160); (c) hamster thymus section, showing $CK18^+$ subseptal and cortical TEC (× 320); (d) $CK18^+$ cortical and medullary TEC in the opossum thymus (× 250). C: cortex, M: medulla.

### Localization of "Stratified" Cytokeratins in Mammalian Thymuses

We also evaluated the localization of cytokeratins typically found in rather complex stratified lining epithelia (Quinlan et al., 1985; Sun et al., 1985). One of the anti-CK MAb used for that, named KL1, recognizes the CK1/CK10 pair, the marker of terminal differentiation in the epidermal epithelium (Viac et al., 1983). Using this reagent, we detected the same distribution pattern of CK expression in both primate thymuses, with the whole thymic epithelium being labeled. Other mammals presented KL1 staining preferentially at the medullary region, with the number of $KL1^+$ cells varying from species to species. These data are summarized in Fig. 4.

In addition to CK1/CK10, we studied the expression of CK13, typical of stratified epithelia of internal organs. In human, Rhesus monkey, and hamster thymuses, subcapsullary–medullary TEC were consistently labeled by the anti-CK13 MAb. This pattern differed in mouse, rabbit and opossum species, in which a medullary restriction of CK13 localization was observed. Conversely, sheep thymic epithelium was totally stained by the anti-CK13 MAb, whereas rat thymic epithelial cells remained negative to this reagent (Fig. 5).

### Immunoblot Detection of Thymic Cytokeratins

As the immunocytochemical reactivity patterns of anti-CK MAb presented an interspecific diversity in the intralobular localization of distinct cytokeratins, it might be possible that the same MAb recognized distinct CK in different species, though these proteins are evolutively conserved (Moll et al., 1982a; Fuchs and Marchuk, 1983;

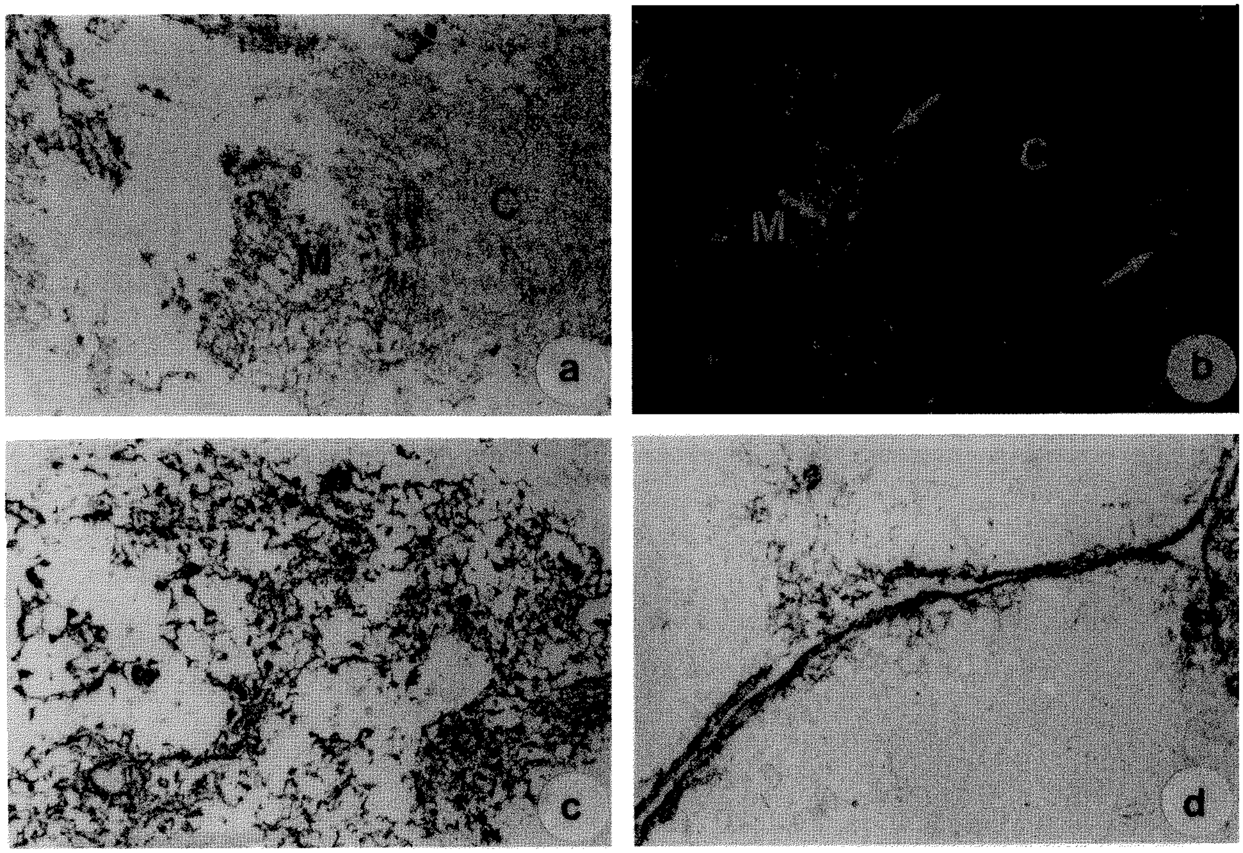

FIGURE 3. CK19-expression pattern in mammalian thymuses: (a) sheep thymus section, with anti-CK19 staining in the whole thymic epithelial network (x 250); (b) rat thymus section showing $CK19^+$ TEC (arrows) in subcapsullary and medullary zones (x 250); (c) Rhesus monkey medullary TEC labeled by the anti-CK19 MAb (x 250); (d) subcapsullary layer of Rhesus monkey thymus labeled with anti-CK19 MAb. C: cortex, M: medulla.

Blumenberg, 1988). Thus, we used a one-dimensional immunoblot assay to approach the molecular specificity of some representative anti-CK MAb in thymic extracts from Rhesus monkey, hamster, rat, rabbit, and sheep. For that, we applied the monoclonal reagents KL1 and anti-CK18, respectively, examples of markers of stratified and simple lining epithelia.

The results obtained with KL1 clearly showed that, although this MAb presented different reactivity patterns *in situ*, it recognized the same CK pair of apparent molecular weight (MW) of 58 and 66 kDa in the various extracts (Fig. 6a). In parallel with these findings, the anti-CK18 MAb reacted with a 45-kDa CK protein band in monkey, rat, and sheep extracts, whereas their *in situ* staining pattern differed among these mammals (Fig. 6b).

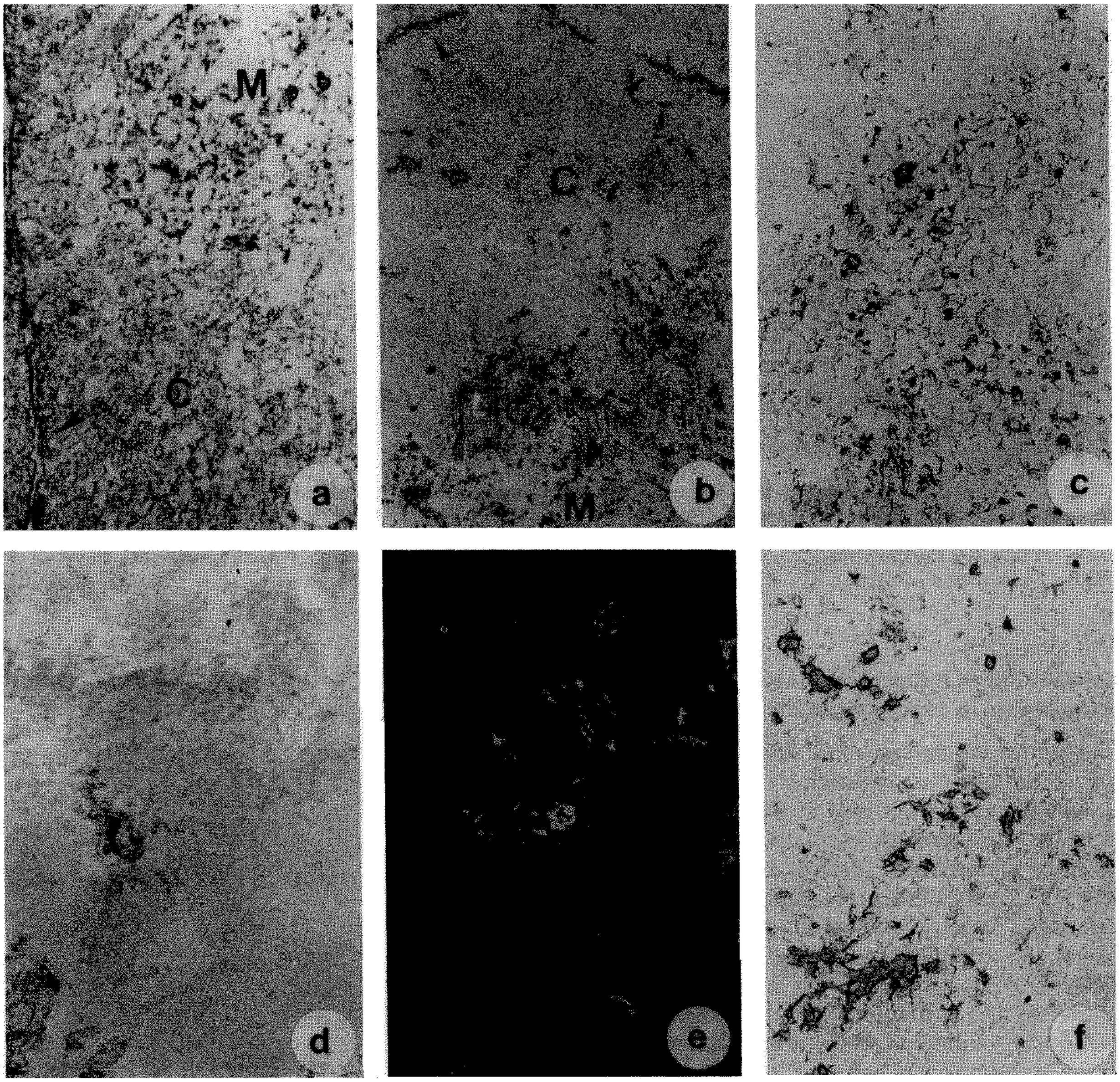

FIGURE 4. KL1 reactivity pattern in mammalian thymuses: (a) Rhesus monkey TEC network totally stained by KL1 (× 160); (b) hamster thymus section, showing subseptal and medullary KL1$^+$ TEC (× 160); (c) rabbit thymic medulla, with the majority of TEC being labeled by KL1 (× 250); KL1$^+$ medullary TEC in thymuses from : (d) sheep (× 250), (e) rat (× 200), and (f) opossum (× 250). C: cortex, M: medulla; arrows indicate KL1$^+$ subseptal TEC.

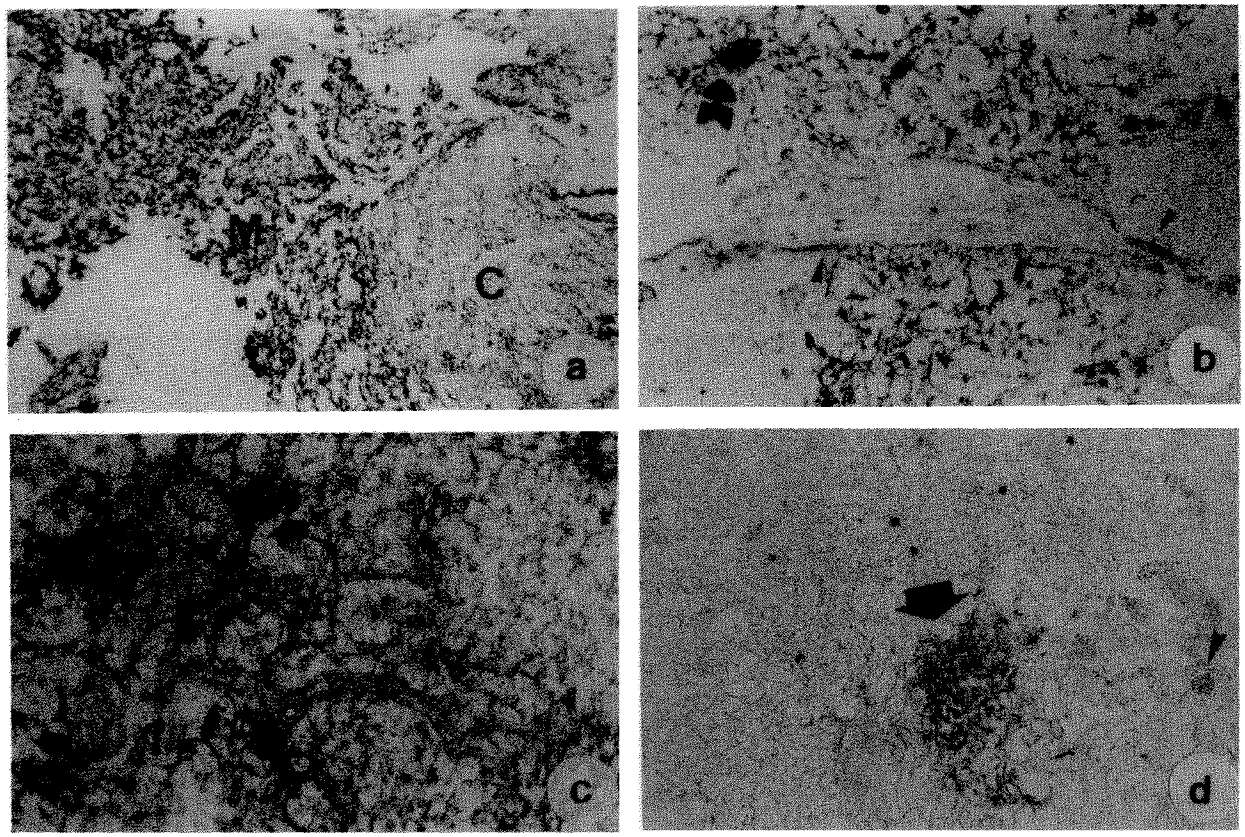

FIGURE 5. CK13-expression pattern in mammalian thymuses: (a) sheep thymus section, showing CK13$^+$ cortical and medullary TEC (× 160); (b) monkey thymus showing CK13 staining in the medullary region, including Hassall's corpuscles (large arrow) and subseptal TEC (short arrow) (× 250); (c) CK-13 labeling of hamster thymic medulla, including Hassall's corpuscles (arrows) (× 400); (d) opossum thymus section, showing CK13$^+$ medullary TEC (large arrow) and CK!3$^-$ Hassall's corpuscles (short arrow) (× 250). C: cortex, M: medulla.

### Distribution of Extracellular Matrix Components in Mammalian Thymuses

Considering the interspecific diversity of the thymic epithelium, as defined by the cytokeratin expression, we carried out a comparative analysis of thymic extracellular matrix proteins in the different mammalian species. We chose typical basement membrane proteins, namely, laminin, fibronectin, and type-IV collagen, as representatives of a distinct component of the thymic microenvironment, yet being largely produced by the thymic epithelium. Additionally, we had previously described their topographic distribution in human and mouse thymuses (Berrih et al., 1985; Lannes-Vieira et al., 1991).

In contrast to the CK interspecific diversity, the distribution of extracellular matrix proteins was strikingly similar in mammals, with labeling

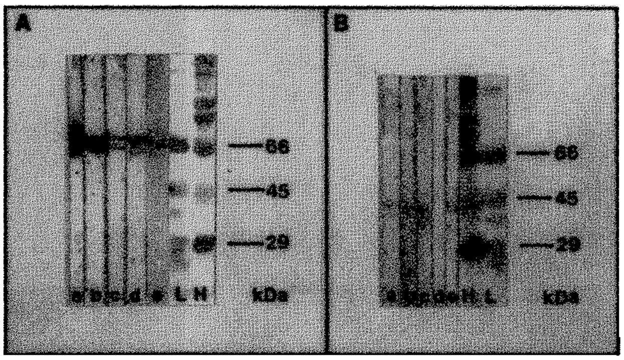

FIGURE 6. Cytokeratins recognized by (A) KL1 and (B) anti-CK18 MAb in one-dimensional immunoblotassay of thymic extracts derived from (a) Rhesus monkey, (b) hamster, (c) rat, (d) rabbit, and (e) sheep. L: low-molecular-weight standard markers, H: high-molecular-weight standard markers.

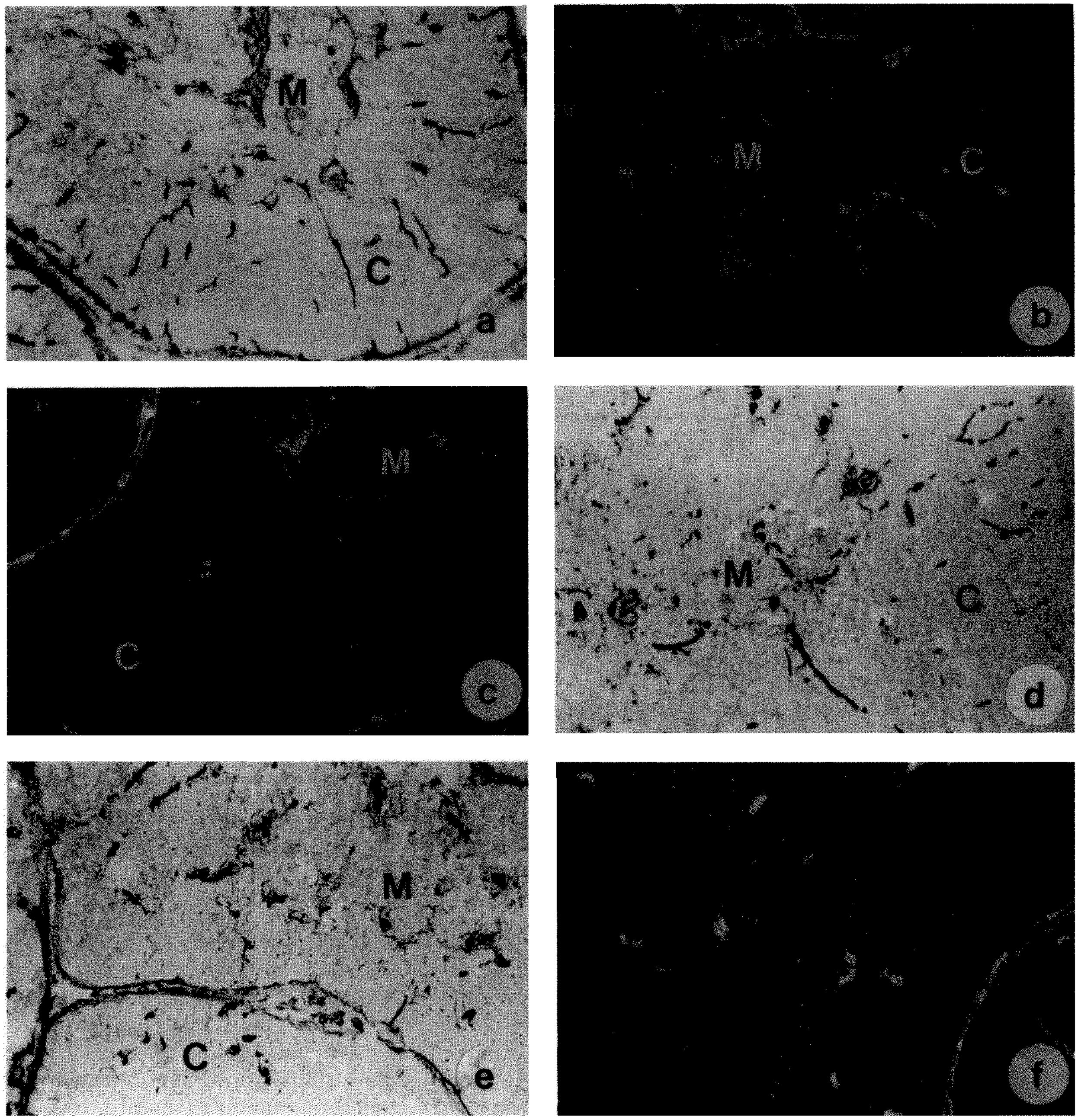

FIGURE 7. Distribution pattern of basement membrane components in mammalian thymuses: (a) hamster thymus section, showing the fibronectin localization in basement membranes of septa and trabeculae, and a fine medullary meshwork (× 160); (b) human thymus section, revealing fibronectin staining in septum and medullary network (× 320); (c) short and thin laminin fibers observed in cortical and medullary regions of rat thymus (× 320); (d) laminin expression in trabeculae, perivascular spaces, and medullary fibers in monkey thymus (× 160); (e) sheep thymus section, showing septa, trabeculae, and medullary fibers labeled by the anti-type-IV collagen antibody (× 160); (f) type-IV collagen staining of rat thymus, showing the particular short and thin fibers (× 320). C: cortex, M: medulla.

being restricted to the typical basement membranes adjacent to capsule, septa, and blood vessels, together with a fine medullary network (Fig. 7). The rat thymus was the only one that

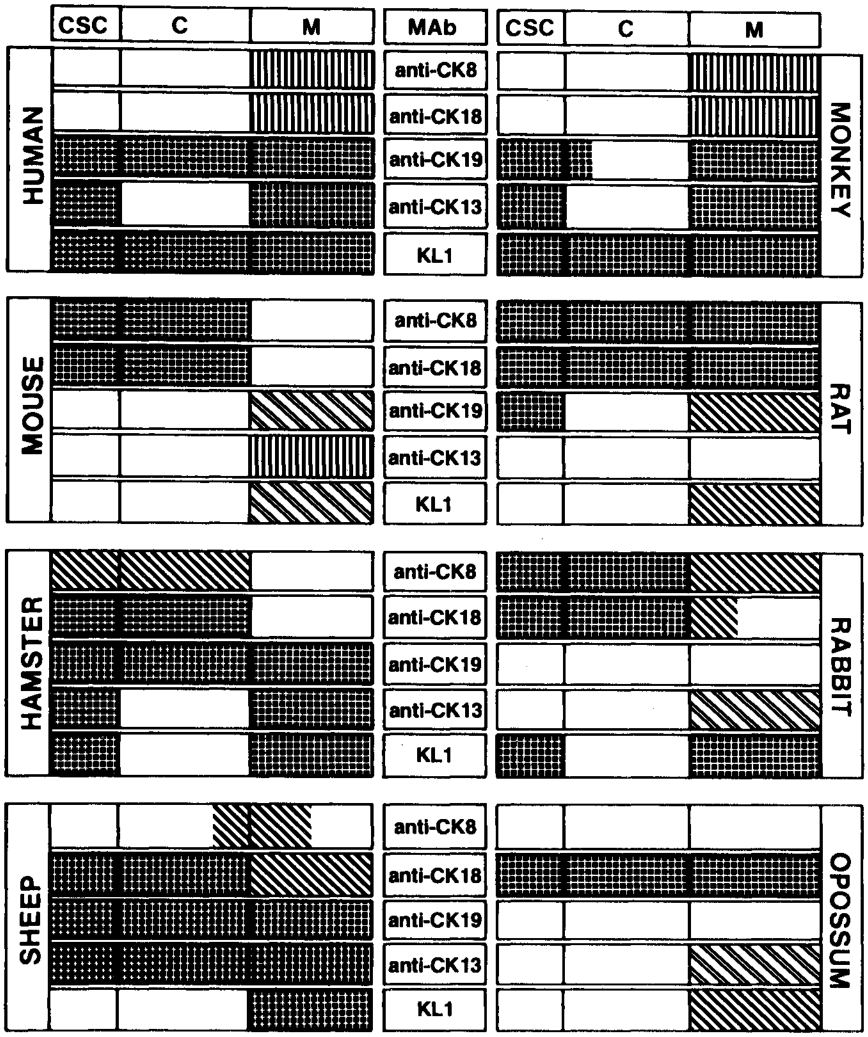

FIGURE 8. The interspecific diversity of cytokeratin expression pattern in mammalian thymuses. ▇: all cells of the region being labeled; ▥: many cells of the region being labeled; ▨: few cells labeled; ▧: very few labeled cells, and ▭: no labeled cells in the region.

diverged, because it presented short and thin fibronectin, type-IV collagen, and laminin fibers both at the cortical and medullary regions.

### DISCUSSION

The heterogeneity of the thymic microenvironment, and in particular of its epithelium component, is already largely accepted. Nonetheless, an interspecific diversity in the localization of molecules expressed by TEC has been much less investigated. In this regard, the present work brings consistent data revealing that the topography of TEC-expressing specific cytokeratins varies amongst mammalian species belonging to distinct phylogenetic orders (summarized in Fig. 8). This was initially suspected by our previous data on CK distribution in the mouse and human thymuses (Savino and Dardenne, 1988a, 1988b), and more recently by Colic et al. (1990) studying other mammalian species. Nevertheless, these findings were strictly based on immunohistochemistry, thus being unable to discard the possibility of epitope cross reactivity among distinct cytokeratins in different species. Actually, we showed herein that the same specific molecular weight cytokeratins could be evidenced with KL1 and anti-CK18 MAb in thymic extracts from several species, independently of the diversity in their immunohistochemically defined intrathymic localization. These data suggest that, for a variety of mammalian species, there is a topographical diversity of CK expression, or at least

TABLE 1
Classification of Mammalian Thymuses for Cytokeratin Expression Patterns Based on the "Clusters of Thymic Epithelial Staining" Nomenclature[a]

| | Anti-CK8 | Anti-CK18 | Anti-CK19 | Anti-CK13 | KL1 |
|---|---|---|---|---|---|
| Human | XX | XX | I | II | I |
| Monkey | XX | XX | II.A | II | I |
| Hamster | III | III | I | II | II |
| Mouse | III | III | V.C | XX | V.A |
| Rat | I | I | V.E | – | V.C |
| Rabbit | III.A | XX | – | XX | II |
| Sheep | XX | I | I | I | IV |
| Opossum | – | I | – | V.C | V.C |

[a]I–XX: classification according to CTES nomenclature (Kampinga et al., 1989); –: negative.

of CK epitopes. The approach of *in situ* hybridization using CK-specific mRNA or cDNA probes, or the use of polyclonal antibodies produced against synthetic CK peptides (Roop et al., 1984; Ruggiero et al., 1990) would be useful to clarify this question. Nonetheless, even if the integral CK molecules were not heterogeneously distributed among species, a CK epitope diversity was definitely detected. Interestingly, these epitopes could be functional, because modulations of CK staining patterns were observed in many physiological and pathological situations (Meireles de Souza and Savino, 1993).

The present work also demonstrates that the pattern of CK expression by the thymic epithelium is complex and rather unique, with cytokeratins typically found in simple or stratified epithelia being coexpressed. It is noteworthy that the colocalization of CK8, CK18, CK19, CK1/CK10 pair, and CK13 was exclusively found in this epithelium (compare to data presented in Quinlan et al., 1985). Whereas the CK1/CK10 pair is expressed by highly keratinized epithelia, as the epidermis, the CK13 is typical of many internal stratified epithelia, like the esophagus, trachea, and tongue, and in general are mutually exclusive. The complexity and uniqueness of the thymic epithelium CK expression were further emphasized by Heid et al. (1988) describing the presence of hair-type keratins in the thymus. Taken together, the previous findings suggest that the general classification of epithelial tissues based on their pattern of cytokeratin expression cannot be applied to the thymus. The interspecific diversity of CK expression, when analyzed in terms of clusters of thymic epithelial-cell staining, grouped the CK-defined TEC subsets in different CTES (Kampinga et al., 1989), depending on the mammalian species analyzed, as depicted in Table 1. Thus, CK8$^+$ TEC are classified as group I in rat, group II in mouse and hamster, and group XX in primates. Yet, anti-TEC MAb can be classified in different CTES groups according to the species (Kampinga et al., 1989). This point indicates that the applicability of CTES nomenclature is rather restrict.

In spite of interspecific diversity of cytokeratin localization within thymic lobules, important similarity was observed in species belonging to the same mammalian order in the phylogenetic tree. This concept can be inferred from Fig. 8. In particular, human and Rhesus monkey diverged only in the CK19$^+$ TEC distribution. Moreover, the anti-CK8, anti-CK18, and KL1 MAb reactivity patterns seemed to be exclusive features of this order.

We also observed a compartmentalization of the same CK in cortical or medullary regions in different mammals. Particularly, we verified that TEC recognized by KL1 or anti-CK13 MAb (and in many instances by the anti-CK19 MAb) were predominantly found in medulla. Actually, in the cases when these MAb labeled the whole (or most of) medullary region, subcapsullary TEC were also stained. This characteristic is shared with many other anti-TEC MAb (Haynes, 1984; de Maagd et al., 1985; Takacs et al., 1987; Kampinga and Aspinall, 1990; Izon and Boyd, 1990), suggesting profound similarities in subcapsullary–medullary TEC that might be related to a hypothetic common embryonic origin (Crouse et al., 1985; Lobach and Haynes, 1987). Nevertheless, studies on cytokeratin expression did not clarify the question of whether the thymic epithelium is derived from endoderm or endoectoderm (Le Douarin et al., 1984; von Gaudecker, 1986; Lampert and Ritter, 1988). In fact, the localization of the same CK in different thymic regions in diverse species may imply that anti-CK MAb are not appropriate embryonic markers.

In contrast to cytokeratin expression data, the study of the extracellular matrix molecule localization in mammalian thymuses revealed a conserved pattern, except for the rat thymus. This conservation suggests that these molecules may be involved in important events of thymic physiology, as recently proposed (Savino and Lannes-Vieira, 1991).

TABLE 2
General Characteristics of Anti-Cytokeratin Antibodies and Their Labeling Patterns in the Mouse Thymus

| Antibody (origin) | Immunogen | Specificity | Recognized TEC[a] in mice[b] |
|---|---|---|---|
| HTK (Biosoft) | Human epidermis | Various CK[a] | Whole TEC network |
| KL4 (Immunotech) | Human epidermis | Various CK | Whole TEC network |
| RPN1166 (Amersham) | PtK1 cells (rat canguru) | CK8 | Cortical TEC |
| RPN1160 (Amersham) | PtK1 cells (rat canguru) | CK18 | Cortical TEC |
| RPN1165 (Amersham) | SV40-transformed human keratinocyte | CK19 | Few medullary TEC |
| $K_s$13.1 (Progen) | CK13 isolated from human esophage | CK13 | Not previously tested |
| KL1 (Immunotech) | Human epidermis | CK1/CK10 | Few medullary TEC |

[a]TEC: thymic epithelial cells; CK=cytokeratin.
[b]References: Nicholas et al. (1985); Savino and Dardenne (1988a).

Lastly, if we consider these results together with the concept that thymocyte differentiation appears to occur in a similar way in different species (Fowlkes and Pardoll, 1989), and that functional TEC subsets might be related to diverse events of T-lymphocyte maturation, we are impelled to think that anti-CK and anti-TEC MAb (yet presenting an interspecific diversity of staining) may not be functional markers of TEC subpopulations. These MAb should be rather regarded as markers of microenvironmental variant series antigens in analogy to the recently proposed species-specific cell-surface antigens—the variant series—of developing thymocytes (Aspinall et al., 1991).

### MATERIALS AND METHODS

### Thymuses

Human thymus biopsies were derived from children aging from 6 months to 4 years that were submitted to surgeries due to cardiac malformations.

Besides human specimens, this study comprised material from different mammalian species, including animals from five phylogenetically different orders, as depicted in Fig. 1. Thymuses from Rhesus monkey (*Macaca mulatta*), hamster (*Mesocricetus auratus*), rat (*Ratus norvegicus*), rabbit (*Oryctolagus cuniculus*), sheep (*Ovis aries*), and opossum (*Didelphis marsupialis*) were provided by the animal facilities of the Oswaldo Cruz Foundation, and C57BL/6 mice (*Mus musculus*) were obtained from the animal house of the Department of Immunology, Sao Paulo University. All animals were young adults.

### Antibodies

A panel of antibodies recognizing different cytokeratins was used. The polyclonal antibody HTK (Biosoft, Paris) and the monoclonal antibody KL4 (Immunotech, Marseille), pan-specific to cytokeratins, proved to label the whole thymic epithelium network (Savino and Dardenne, 1988a). The monoclonal reagent KL1, specific to CK1/CK10 pair (Viac et al., 1983), was purchased from Immunotech (Marseille), whereas other MAb, respectively, monospecific to CK8, CK18 (both typical of simple epithelia), or CK19, were Amersham products (Buckinghamshire, UK). The MAb anti-CK13, marker of esophageal differentiation, was obtained from Progen Biotechnik (Heidelberg). General characteristics of these antibodies recognizing distinct thymic epithelial cells are summarized in Table 2.

Extracellular matrix components of the thymic microenvironment were analyzed with rabbit antisera specific to type-IV collagen, fibronectin, or laminin (Grimaud et al., 1980), purchased from Institute Pasteur (Lyon, France). The distribution patterns of the molecules specifically detected by these antibodies in the human and mouse thymuses were previously reported (Berrih et al., 1985; Lannes-Vieira et al., 1991).

We further used biotinilated or fluoresceinated goat antisera as secondary antibodies. Biotinilated immunesera anti-mouse Ig (GAM/BIOT) and anti-rabbit Ig (GAR/BIOT) were obtained from Amersham, and fluoresceinisothiocyanate-coupled anti-mouse Ig serum (GAM/FITC) and anti-rabbit Ig goat serum bound to tetramethylrhodamineisothiocyanate (GAR/TRITC) were purchased from Biosys (Compiegne, France).

### Immunohistochemistry

Acetone-fixed, 4-$\mu$m thick thymus frozen sections were subjected to indirect immunofluorescence or immunoperoxidase assays according to previous descriptions (Nicolas et al., 1985). Specimens were incubated for 1 hr with a certain primary antibody and washed in PBS. With regard to the immunofluorescence technique, primary antibodies were revealed with appropriate GAM/FITC or GAR/TRITC, incubated for 1 hr. After washing in PBS, slides were mounted in glycerol/PBS and examined through a Leitz Ortoplan fluorescence microscope (Wetzlar, Germany). The rabbit serum pan-specific to cytokeratins and the mouse MAb monospecific to certain cytokeratins were used in double labeling assays.

For streptavidin-biotin immunoperoxidase staining, specimens were subjected to biotinilated secondary antibodies (GAM/BIOT or GAR/BIOT) for 1 hr, washed in PBS, and then exposed to the streptavidin/peroxidase complex (Amersham, Buckinghamshire, UK) for 1 hr. After washing in PBS, peroxidase activity was revealed by a 10-min incubation with aminoethylcarbazole (Sigma Co., Saint Louis, MO) in the presence of $H_2O_2$. Slides were counterstained with Mayer's hematoxylin and mounted in gelatin/glycerol/fenol.

### Immunoblot Analysis of Cytokeratins

The molecular specificity of anti-CK MAb, namely, anti-CK18 and KL1, representatives of different patterns of TEC labeling, were tested on thymus extracts (including those from monkey, hamster, rat, rabbit and sheep) by one-dimensional immunoblot analysis. Extracts enriched in cytokeratins were obtained by a modification of the technique previously described by Woodcock-Mitchell et al. (1982). Thymic fragments of each species were homogenized in glass potters with 25 mM Tris-HCl (pH 7.2) and 1 mM PMSF at 4°C. Supernatants containing mostly thymocytes were discarded and pellets were subjected to the lysis buffer containing 25 mM Tris-HCl (pH 7.2), 1 mM EDTA, 1 mM EGTA, 1 mM PMSF, and 1% Triton x 100, at 4°C. This suspension was centrifuged at 10,000 g for 40 min at 4°C, the supernatant discarded, and the pellet resuspended in 25 mM Tris-HCl (pH 7.2), 1 mM PMSF at 4°C, being then sonicated for 15 min (Bransonic 2200, Branson Ultrasonics C., Danbury, CN). After one further centrifugation at 10,000 g, the pellet was submitted to a 5-min incubation in Tris-HCl, 1% SDS, 5% 2-mercaptoethanol at 95°C. Extracts were aliquoted and stocked at −20°C.

Protein bands were resolved by SDS-PAGE in 10% acrylamide gels, as originally described by Laemli (1970). Proteins were then blotted onto nitrocellulose sheets (Schleicher and Schuell, Dassel, Germany) according to Towbin et al. (1979). Transfer was checked with Ponceau S Red, and nonspecific binding sites were saturated by 1-hr horizontal agitation with PBS containing 10% fetal calf serum (FCS) and 0.05% Tween 20, at room temperature. Bands were then incubated overnight within a certain anti-CK solution at 4°C. The anti-CK18 was diluted 1:10 in saturation solution and KL1 was diluted 1:200. After washing in PBS/FCS/Tween for 30 min, nitrocellulose sheets were incubated with GAM/BIOT (1:500) for 2 hr, washed, and finally incubated in Streptavidin-peroxidase complex (1:500) for 2 hr. After a further washing, enzyme activity was demonstrated with 3',3-diaminobenzidine (0.3 mg/ml Tris-HCl, pH 7.6) in the presence of 0.03% $H_2O_2$.

### ACKNOWLEDGMENTS

This work was partially supported by the Brazilian Research Council (CNPq) and Commission of the European Communities (CEC).

*(Received July 23, 1992)*

*(Accepted September 18, 1992)*

### REFERENCES

Aspinall R., Kampinga J., and van den Bogaerde J. (1991). T-cell development in the fetus and the invariant series hypothesis. Immunol. Today **12:** 7–10.

Berrih S., Savino W., and Cohen S. (1985). Extracellular matrix of the human thymus: Immunofluorescence studies on frozen sections and cultured epithelial cells. J. Histochem. Cytochem. **33:** 655–664.

Blackman M., Kappler J., and Marrack P. (1990). The role of the T cell receptor in positive and negative selection of development T cells. Science **248:** 1335–1341.

Blumenberg M. (1988). Concerted gene duplications in the two keratin gene families. J. Mol. Evol. **27:** 203–211.

Boyd R., and Hugo P. (1991). An integrated view of the thymic microenvironment. Immunol. Today. **132:** 718–720.

Colic M., Dragojevic-Simic V., Gasic S., and Dujic A. (1990). Interspecies differences in expression of cytokeratin polypeptides within thymic epithelium: A comparative immunohistochemical study. Develop. Comp. Immunol. **14:** 347–354.

Colic M., Matanovic D., Hegedis L., and Dujic A. (1988a). Immunohistochemical characterization of rat thymic nonlymphoid cells. I. Epithelial and mesenchymal components defined by monoclonal antibodies. Immunology **65:** 277–284.

Colic M., Matanovic D., Hegedis L., and Dujic A. (1988b). Heterogeneity of rat thymic epithelium defined by monoclonal anti-keratin antibodies. Thymus **12:** 123–130.

Crouse D.A., Turpen J.B., and Sharp J.G. (1985). Thymic nonlymphoid cells. Surv. Immunol. Res. **4:** 120–134.

de Maagd R.A., Mackenzie W.A., Schuurman H.-J., Ritter M.A., Price K.M., Broekhuizen R., and Kater L. (1985). The human thymus microenvironment: Heterogeneity detected by monoclonal anti-epithelial antibodies. Immunology **54:** 745–754.

Farr A.G., and Brady S.C. (1989). Patterns of keratin expression in the murine thymus. Anat. Rec. **224:** 374–378.

Fowlkes B.J., and Pardoll D.M. (1989). Molecular and cellular events of T cell development. Adv. Immunol. **44:** 207–264.

Fuchs E., and Marchuk D. (1983). Type I and type II keratins have evolved from lower eukaryotes to form the epidermal intermediate filaments in mammalian skin. Proc. Natl. Acad. Sci. USA **80:** 5857–5861.

Grimaud J.A., Druguet M., Peyrol S., Chevalier O., Herbage D., and Elbadrawyn N. (1980). Collagen immunotyping in human liver. J. Histochem. Cytochem. **28:** 11.

Haynes B.F. (1984). The human thymic microenvironment. Adv. Immunol. **36:** 87–142.

Haynes B.F. (1990). Human thymic epithelium and T cell development: Current issues and future directions. Thymus **16:** 143–157.

Heid H.W., Moll I., and Franke W.W. (1988). Patterns of expression of trichocytic and epithelial cytokeratins in mammalian tissues. II. Concomitant and mutually exclusive synthesis of trichocytic and epithelial cytokeratins in diverse human and bovine tissues (hair follicle, nail bed and matrix, lingual papilla, thymic reticulum). Differentiation **37:** 215–230.

Itoh T. (1979). Establishment of an epithelial cell line from rat thymus. Am. J. Anat. **156:** 99–105.

Izon D.J., and Boyd R.L. (1990). The cytoarchitecture of the human thymus detected by monoclonal antibodies. Human Immunol. **27:** 16–32.

Kampinga J., and Aspinall R. (1990). Thymocyte differentiation and thymic microenvironment development in the fetal rat thymus: An immunohistological approach. *In*: Thumus update, 3. The role of the thymus in tolerance induction. Part 2: Current topics and reports from recent meetings, Kendall M.D., and Ritter MA., Eds. (London: Harwood Academic Publishers), pp. 149–186.

Kampinga J., Berges S., Boyd R.L., Brekelmans P., Colic M., van Ewijk W., Kendall M.D., Ladyman H., Nieuwenhuis P., Ritter M.A., Schuurman H.-J., and Tournefier A. (1989). Thymic epithelial antibodies: Immunohistological analysis and introduction of nomenclature. Thymus **13:** 165–173.

Kaneshima H., Ito M., Asai J., Taguchi O., and Hiai H. (1987). Thymic epithelial reticular cell subpopulations in mice defined by monoclonal antibodies. Lab. Invest. **56:** 372–380.

Kendall M.D. (1988). Anatomical and physiological factors influencing the thymic microenvironment. *In*: Thymus update, 1, Kendall M.D. and Ritter M.A., Eds. (London: Harwood Academic Publishers), pp. 27–65.

Kyewski B.A. (1986). Thymic nurse cells: Possible sites of T-cell selection. Immunol. Today **7:** 374–379.

Laemli U.K. (1970). Cleavage of structural proteins during the assembly of the head of bacteriophage T4. Nature **227:** 680.

Lampert I.A., and Ritter M.A. (1988). The origin of the diverse epithelial cells of the thymus: Is there a common stem cell? *In*: Thymus update, 1, Kendall M.D., and Ritter M.A., Eds. (London: Harwood Academic Publishers), pp. 5–25.

Lannes-Vieira J., Dardenne M., and Savino W. (1991). Extracellular matrix components of the mouse thymus microenvironment. Ontogenetic studies and modulation by glucocorticoid hormones. J. Histochem. Cytochem. **39:** 1539–1546.

Le Douarin N.M., Dieterlen-Lièvre F., and Oliver P.D. (1984). Ontogeny of primary lymphoid organs and lymphoid stem cells. Am. J. Anat. **170:** 261–299.

Lobach D.F., and Haynes B.F. (1987). Ontogeny of the human thymus during fetal development. J. Clin. Immunol. **7:** 81–97.

MacDonald F., Ed. (1984) The encyclopedia of mammals (London: Allen & Unwin).

Meireles de Souza L.R., and Savino, W. (1993). Modulation of cytokeratin expression in the hamster thymus: Evidence for a plasticity of the thymic epithelium. Dev. Immunol.

Mizutani S., Watt S.M., Robertson D., Hussein S., Healy L.E., Furley A.J.W., and Greaves M.F. (1987). Cloning of human thymic subcapsular cortex epithelial cells with T-lymphocyte binding sites and hemopoietic growth factor activity. Proc. Natl. Acad. Sci. USA **84:** 4999–5003.

Moll R., Franke W.W., and Schiller D.L. (1982). The catalog of human cytokeratins: Patterns of expression in normal epithelia, tumors and cultured cells. Cell **31:** 11–24.

Naquet P., Lepesant H., Luxembourg A., Brekelmans P., Devaux C. and Pierres M. (1989). Establishment and characterization of mouse thymic epithelial cell lines. Thymus **13:** 217–224.

Nicolas J.-F., Reano A., Kaiserlian D., and Thivolet J. (1986). Epithelial cell heterogeneity in the guinea pig thymus: Immunohistochemical characterization of four thymic epithelial subsets defined by monoclonal anti-keratin antibodies. Eur. J. Immunol. **16:** 457–464.

Nicolas J.-F., Savino W., Reano A., Viac J., Brochier J., and Dardenne M. (1985). Heterogeneity of thymic epithelial cell (TEC) keratins. Immunohistochemical and biochemical evidence for a subset of highly differentiated TEC in the mouse. J. Histochem. Cytochem. **33:** 687–694.

Nieburgs A.C., Picciano P.T., Korn J.H., McCalister T., Allred C., and Cohen S. (1985). *In vitro* growth and maintenance of two morphologically distinct populations of thymic epithelial cells. Cell. Immunol. **90:** 439–450.

Potworowski E.F., Turcotte F., Beauchemin C., Hugo P., and Zelechowska M.G. (1986). Establishment and characterization of a thymic medullary epithelial cell clone. In Vitro Cell. & Develop. Biol. **22:** 557–560.

Quinlan R.A., Schiller D.L., Hatzfeld M., Achtstatter T., Moll R., Jorcano J.L., Magin T.M., and Franke W.W. (1985). Patterns of expression and organization of cytokeratin intermediate filaments. Ann. N.Y. Acad. Sci. **455:** 282–305.

Roop D.R., Cheng C.K., Titterington L., Meyers C.A., Stanley J.R., Steinert P.M., and Yuspa S.H. (1984). Synthetic peptides corresponding to keratin subunits elicit highly specific antibodies. J. Biol. Chem. **259,** 8037–8040.

Ruggiero P., Petracca R., and Leoncini P. (1990). Immunolocalization of intermediate filaments proteins using antibodies to synthetic peptides. J. Histochem. Cytochem. **38:** 993–999.

Savino W., and Dardenne M. (1988a). Developmental studies on the expression of monoclonal antibody defined cytokeratins by thymic epithelial cells from normal and autoimmune mice. J. Histochem. Cytochem. **36:** 1123–1128.

Savino W., and Dardenne M. (1988b). Immunohistochemical studies on a human thymic epithelial cell subset defined by the anticytokeratin 18 monoclonal antibody. Cell Tissue Res. **254:** 225–231.

Savino W., and Lannes-Vieira J. (1991). Is there a role for extracellular matrix in thymus physiology and pathology? Mem. Inst. Oswaldo Cruz (Rio de Janeiro) **85 (Suppl. IV):** 109–115.

Schmitt D., Zambruno G., Staquet M.-J., Dezutter-Dambuyant C., Ohrt C., Brochier J., and Thivolet J. (1987). Antigenic thymus-epidermis relationships. Reactivity of a panel of anti-thymic cell monoclonal antibodies on human keratinocytes and Langerhans cells. Dermatologica **175:** 109–120.

Schuurman H.-J. (1988). Biological functions of the thymic microenvironment. *In*: Thymus Update, 1, Kendall M.D., and Ritter M.A., Eds. (London: Harwood Academic Publishers), pp. 67–99.

Sprent J., Lo D., Gao E.-K., and Ron Y. (1988). T cell selection in the thymus. Immunol. Rev. **101:** 173–190.

Sun T.-T., Tseng S.C.G., Huang A.J.-W., Cooper D., Schermer A., Lynch M.H., Weiss R., and Eichner R. (1985). Monoclonal antibody studies of mammalian epithelial keratins: A review. Ann. N.Y. Acad. Sci. **455:** 307–329.

Takacs L., Savino W., Monostori E., Ando I., Bach J.-F., and Dardenne M. (1987). Immunohistologic analysis of the pathologic microenvironment. J. Immunol. **138:** 687–699.

Towbin H., Staehelin T., and Gordon J. (1979). Electrophoretic transfer of proteins from polyacrylamide gels to nitrocellulose sheets: Procedure and some applications. Proc. Natl. Acad. Sci. USA **76:** 4350–4354.

van Ewijk W. (1991). T-cell differentiation is influenced by thymic microenvironments. Ann. Rev. Immunol. **9:** 591–615.

van Vliet E., Melis M., and van Ewijk W. (1984). Monoclonal antibodies to stromal cell types of the mouse thymus. Eur. J. Immunol. **14:** 524–529.

Viac J., Reano A., Brochier J., Staquet M.-J., and Thivolet J. (1983). Reactivity pattern of a monoclonal antibody (KL1). J. Invest. Dermatol. **81:** 351–354.

von Boehmer H., Teh H.S., and Kisielow P. (1989). The thymus selects the useful, negects the useless and destroys the harmful. Immunol. Today **10:** 57–61.

von Gaudecker B. (1986). The development of the human thymus microenvironment. Cur. Top. Pathol. **75:** 1–41.

Woodcock-Mitchell J., Eichner R., Nelson W.G ., and Sun T.-T. (1982). Immunolocalization of keratin polypeptides in human epidermis using monoclonal antibodies. J. Cell Biol. **95:** 580–587.

